# Pediatric COVID-19 Risk Factors in Southeast Asia-Singapore and Malaysia: A Test-Negative Case–Control Study

**DOI:** 10.4269/ajtmh.21-1000

**Published:** 2022-02-15

**Authors:** Judith Ju Ming Wong, Chin Seng Gan, Sanghvi Heli Kaushal, Soo Lin Chuah, Rehena Sultana, Natalie Woon Hui Tan, Kah Peng Eg, Koh Cheng Thoon, Jan Hau Lee, Chee Fu Yung

**Affiliations:** ^1^ Children’s Intensive Care Unit, Department of Pediatric Subspecialties, KK Women’s and Children’s Hospital, Singapore;; ^2^ Duke–National University of Singapore (NUS) Medical School, Singapore;; ^3^ SingHealth Duke–NUS Global Health Institute, Singapore;; ^4^ Pediatric Intensive Care Unit, Department of Pediatrics, University Malaya Medical Centre, University of Malaya, Kuala Lumpur, Malaysia;; ^5^ Yale–NUS College Singapore;; ^6^ Center for Quantitative Medicine, Duke–NUS Medical School, Singapore;; ^7^ Infectious Disease Service, Department of Pediatrics, KK Women’s and Children’s Hospital, Singapore;; ^8^ Respiratory Medicine, Department of Pediatrics, University Malaya Medical Centre, University of Malaya, Kuala Lumpur, Malaysia;; ^9^ Lee Kong Chian School of Medicine, Imperial College, Nanyang Technological University, Singapore

## Abstract

There is a scarcity of population-level data of pediatric COVID-19 infection from Southeast Asia. This study aims to describe and compare epidemiological, clinical, laboratory and outcome data among pediatric COVID-19 cases versus controls in two neighboring countries, Singapore and Malaysia. We used a test-negative case–control study design recruiting all suspected COVID-19 cases (defined by either clinical or epidemiological criteria) from January 2020 to March 2021 admitted to two main pediatric centers in Singapore and Malaysia. Data were collected using a standardized registry (Pediatric Acute and Critical Care COVID-19 Registry of Asia). The primary outcome was laboratory-confirmed COVID-19. Univariate and multivariable logistic regression analysis was used to determine factors associated with COVID-19. This study included 923 children with median age of 4 (interquartile range 2–9) years. Of these, 35.3% were COVID-19 cases. Children with COVID-19 were more likely to be asymptomatic compared with controls (49.4 versus 18.6%; *P* < 0.0001). They were also less likely to develop respiratory complications, such as bronchitis or pneumonia, or organ dysfunction. Four (1.2%) of our COVID-19 patients required respiratory support compared with 14.2% of controls needing respiratory support. COVID-19 cases tended to have lower neutrophil count but higher hemoglobin compared with controls. There were no reported deaths of COVID-19 infection; in contrast, 0.7% of the control group died. In the multivariable analysis, older age, travel history, and close contact with an infected household member were associated with COVID-19 infection. This study shows that the majority of pediatric COVID-19 cases were of lesser severity compared with other community acquired respiratory infections.

## INTRODUCTION

Severe acute respiratory syndrome coronavirus 2 (SARS-CoV-2) emerged in December 2019 in Wuhan, China, and has affected millions of adults and children alike. ^1^ Singapore and Malaysia identified their first cases on January 24 and 25, 2020, respectively. ^2^^,^ ^3^ COVID-19 had caused significant socioeconomic, political, and health impact across Southeast Asia since the first quarter of 2020. ^4^ According to the Human Development Index, Singapore and Malaysia were ranked within the top three most prepared Southeast Asian countries, with Singapore claiming the top-ranking and Malaysia the third. ^4^ During this pandemic, mitigation measures used by both countries included active case detection through mass testing and contact tracing, case isolation, and nonpharmaceutical public health interventions such as mask wearing, social-distancing measures, school closures, travel restrictions, and national lockdowns. ^5^^–^ ^8^

Since the initial phases of the pandemic, there was an apparent effect of age on the severity of infection, with children and adolescents having considerably fewer and milder COVID-19 infections compared with adults. ^9^ An early retrospective series of pediatric COVID-19 in China (*N* = 2,135) found that COVID-19 manifestations in children were largely mild, although 5.8% of this cohort still developed critical COVID-19 infection, this proportion was considerably lower than that of the adult cohort (18.5%). ^10^ In a meta-analysis including 3,600 pediatric COVID-19 patients, 90% were estimated to be diagnosed with asymptomatic or mild symptoms, whereas 6.7% of the cases were severe, often associated with infants with preexisting underlying conditions (28 of 37 [77%] hospitalized patients), such as cardiovascular disease, immunosuppression, blood disorders, endocrine disorders, and neurodevelopmental conditions. ^11^ This reduced susceptibility in the young may be related to several factors, including the lower expression/affinity or variants of angiotensin-converting enzyme 2 receptor, a receptor for the SARS-CoV-2 spike protein, ^12^^–^ ^14^ and a lower prevalence of comorbidities associated with severe COVID-19 (e.g., cardiovascular disease, diabetes, renal failure). ^15^ Nevertheless, children of all ages are susceptible to contracting and transmitting COVID-19. ^16^ Despite having milder symptoms, the viral load in children was found to be comparable to adults. ^17^ To date, most reports of pediatric COVID-19 were infected through close contact with infected household members or were driven by large community outbreaks. Although large-scale transmission in schools has not been shown to drive community transmission, deescalation of school mask-wearing requirements may result in increased community transmission. ^18^^–^ ^21^

With limited existing data on pediatric COVID-19 infections, it is important to obtain data specifically in the Southeast Asian region with its unique socioeconomic composition. ^22^^–^ ^24^ Differences in COVID-19 infection rates and outcomes are potentially influenced by genetic factors, cultural practices, environmental exposures, or combinations of biological and social factors. ^22^^,^ ^25^ Identifying similarities and differences between local or regional or international patterns and risks factors of pediatric COVID-19 infection is essential to guide clinical management of suspected cases and public health policies such as reopening schools, managing community spread, and optimizing healthcare resources. This understanding is also crucial in informing the risk–benefit of COVID-19 vaccination in children. This study aims to identify epidemiological, clinical, and laboratory related risk factors for COVID-19 in children from Malaysia and Singapore, two countries that are closely linked in terms of ethnicity and culture, geographic proximity, and economic factors as well as pediatric public health parameters such as infant mortality rate, childhood immunization coverage, access to healthcare, and education.

## METHODS

### Study design.

We conducted a test-negative case–control study from January 2020 to March 2021 in two large pediatric tertiary centers, KK Women and Children’s Hospital in Singapore and University Malaya Medical Center in Kuala Lumpur, Malaysia. These centers are part of the Pediatric Acute and Critical Care COVID-19 Registry of Asia, which is a registry (clinicaltrial.gov registration NCT04395781) within the Pediatric Acute and Critical Care Medicine Asian Network. All children and infants from birth to 16 years were admitted fulfilling the suspect criteria (meeting any one of the clinical or epidemiological criteria; supplemental material, Table 1)] for COVID-19 who underwent SARS-CoV2 reverse transcriptase polymerase chain reaction (RT-PCR). Cases were defined by positive nasopharyngeal aspirate for COVID-19 nucleic acid RT-PCR. Controls were defined by testing negative for COVID-19 nucleic acid RT-PCR. Epidemiological, clinical, laboratory, and outcomes data were extracted at participating sites and anonymized data was entered into a secure centralized database set up using Research Electronic Data Capture system (REDCAP) by the main coordinating center in Singapore. ^26^ Outcome data was captured upon discharge from the hospital. A multiplex respiratory pathogen PCR was performed when clinically indicated in both confirmed cases or negative controls to identify co-infections. Reporting was conducted in compliance with the STROBE (Strengthening the Reporting of Observational Studies in Epidemiology) guidelines. ^27^ This study received approval from the respective institutional review boards, and a waiver of consent was granted by both participating hospitals.

**Table 1:** Summary of baseline data in COVID-19 cases and controls

Demographics	COVID-19 (*N* = 326)	Control (*N* = 597)	Total (*N* = 932)	*P* value
Age, years	7 (3,12)	3 (1,7)	4 (2,9)	< 0.0001
Weight, kg	20.6 (11.8–37.8)	13.6 (9.6–21.1)	15 (10.2–26.7)	< 0.0001
Male gender	168 (51.5)	324 (54.3)	492 (53.3)	0.4256
Infants	21 (6.4)	106 (17.8)	127 (13.9)	< 0.0001
Breastfeeding	10 (58.8)	68 (78.2)	78 (75.0)	0.0922
Positive travel history	161 (49.5)	143 (24.0)	304 (33.0)	< 0.0001
Exposure to confirmed case	271 (83.9)	169 (28.3)	440 (47.8)	< 0.0001
Healthcare	5 (1.5)	11 (1.8)	16 (1.7)	0.7312
School	2 (0.6)	17 (2.8)	19 (2.1)	0.0223
Household	259 (79.4)	131 (21.9)	390 (42.3)	< 0.0001
Others	12 (3.7)	13 (2.2)	25 (2.7)	0.1787
Comorbidity	32 (9.8)	168 (28.2)	200 (21.7)	< 0.0001
Cardiovascular	2 (0.6)	12 (2.0)	14 (1.5)	
Respiratory	6 (1.8)	87 (14.6)	93 (10.1)	
Neurology	2 (0.6)	24 (4.0)	26 (2.8)	
Hematology/oncology	4 (1.2)	12 (2.0)	16 (1.7)	
Renal	0 (0.0)	2 (0.3)	2 (0.2)	
Gastrointestinal	1 (0.3)	5 (0.8)	6 (0.7)	
Others	17 (5.2)	26 (4.4)	43 (4.7)	

Categorical and continuous variables are expressed as counts (percentages) and median (interquartile range), respectively. *P* values are based on chi-square test and Mann-Whitney *U* test for categorical and continuous variables, respectively.

### Outcome assessment.

The primary outcome was laboratory-confirmed COVID-19 infection as defined earlier. Severity of COVID-19 cases were classified into four groups: mild (included fever, sore throat, cough and/or myalgia, no dyspnea), moderate (included fever, dyspnea, and/or chest imaging consistent with COVID-19 pneumonia with no change from baseline respiratory support requirement), severe (included fever, dyspnea, and/or chest imaging consistent with COVID-19 pneumonia, with new or increased supplemental O_2_ requirement and/or ventilatory support requirement), and critical (included respiratory failure requiring mechanical ventilation, acute respiratory distress syndrome, shock/systemic inflammatory response syndrome, and/or multiorgan failure) according to the WHO definition. ^28^ We also reported all laboratory-confirmed viral and bacterial infections, hospital duration, final respiratory-related diagnosis, respiratory support and complications, supportive therapies, and organ dysfunction at hospital discharge.

### Statistical analysis.

All variables were summarized in terms of the aforementioned case–control categories. Categorical and continuous variables were presented as counts (percentages) and median (interquartile range), respectively. Association between case–control categories and other categorical variables were evaluated using the chi-square test, whereas the association between continuous variables were tested using the Mann-Whitney *U* test. All laboratory test results collected longitudinally over days 1 to 7, 10, and 14 of admission and were analyzed with repeated-measures mixed-models (repeated analysis of variance), which accounts for the dependence among repeated measurements on the same patient. Mixed models for continuous data were used, age and time by case–control categories interaction as fixed effects, and time as a random effect with a variance components variance–covariance matrix. The estimation method was based on a maximum likelihood technique and the variance–covariance matrix of the parameter estimates computed using a sandwich (empirical) estimator. Estimates from the model was reported as mean with 95% confidence interval (95% CI). Random effects univariate and multivariable logistic regression model with center as random effects was used to determine exposures present at admission that was associated with the primary outcome; covariates chosen for the multivariable model was determined by univariate *P* value. A priori–determined year of admission (2020 versus 2021) was also included to assess for any changes in view of increased detection of new circulating COVID-19 variants of concern in 2021. Quantitative association from univariate and multivariable logistic regression analysis was expressed as unadjusted odds ratio (OR) and adjusted OR (aOR) with 95% CI, respectively. All tests were two sided, and *P* < 0.05 was considered statistically significant. All analysis was performed in SAS v9.4 (SAS Institute, Cary, NC; PROC MIXED & PROC GLIMMIX).

## RESULTS

**Figure 1.**
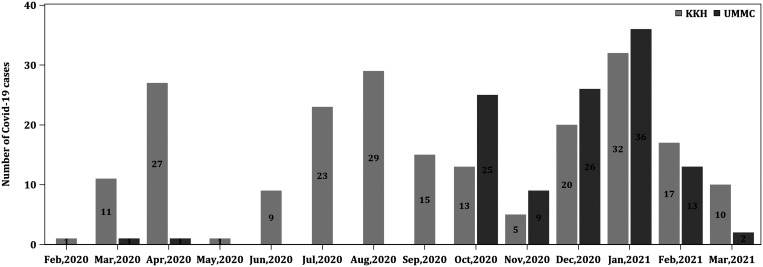
Epidemic curve of the pandemic in children in Singapore and Malaysia. KKH = KK Women’s and Children’s Hospital; UMMC = University Malaya Medical Center.

A total of 923 patients meeting suspect case criteria were included in this analysis over the recruitment period. Of these, 326 of 923 (35.3%) were confirmed cases, whereas the remaining were negative controls. There were 213 of 326 (65.3%) cases from Singapore and 113 of 326 (34.7%) cases from Malaysia. The epidemic curve of the pandemic in children in Singapore and Malaysia respectively is shown in Figure 1.

Confirmation of COVID-19 infection was performed via RT-PCR in all patients. The median (interquartile range) age of COVID-19 cases was older compared with controls: mean 7 (3–12) years versus 3 (1–7) years; *P* < 0.001 (Table 1). Among the positive cases, infants made up only 6.4% of cases (21 of 326). There were no gender differences observed in COVID-19 cases and controls. Exposure to an infected household contact was the most common source of COVID-19 transmission to children (259 of 326 [79.4%]), followed by exposure in school (2 of 326 [0.6%]) or healthcare settings (5 of 326 [1.5%]). The proportion of patients with comorbidity was higher in the control group (28.2%) than the COVID-19 group (9.8%).

**Figure 2.**
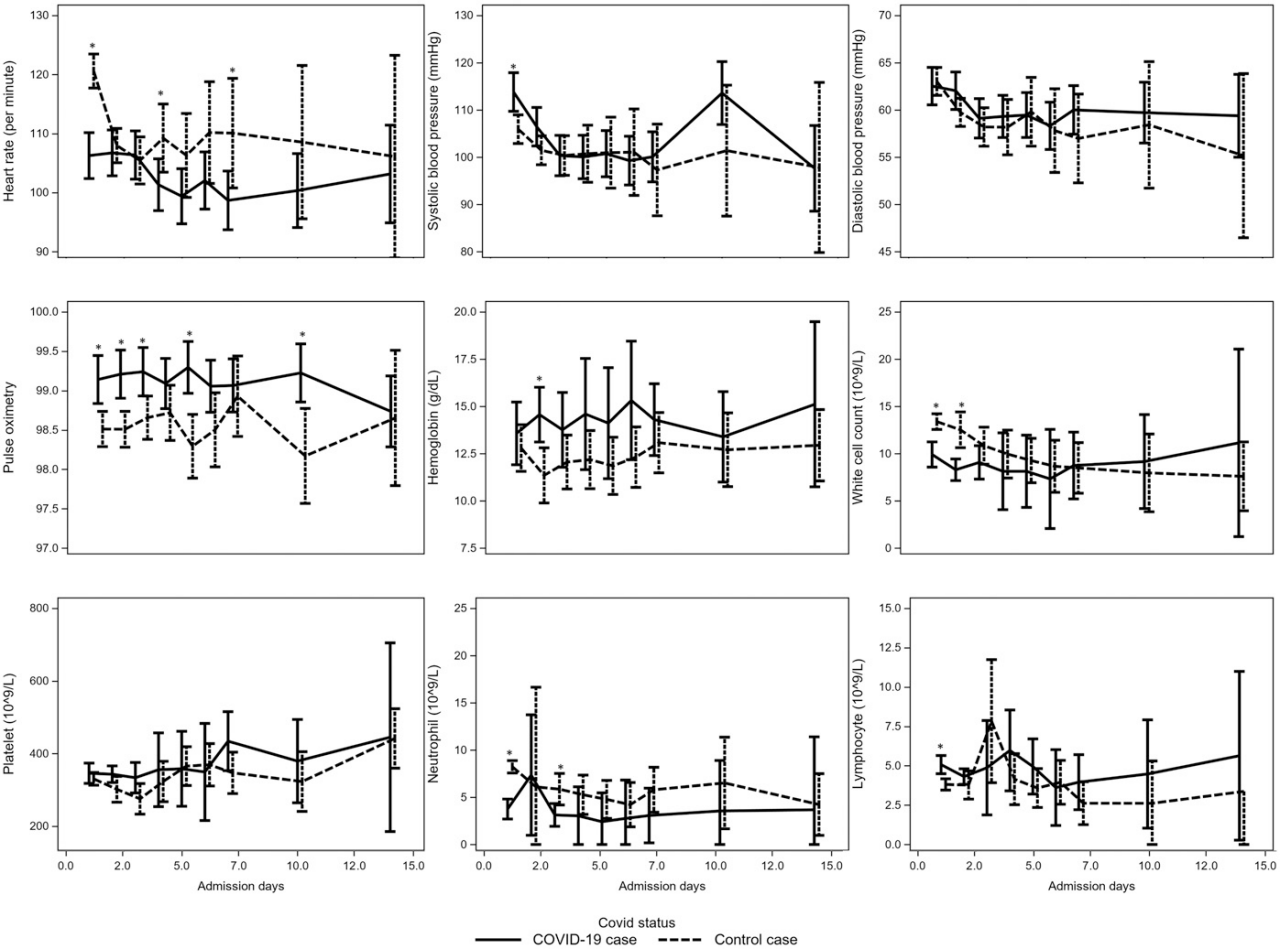
Temporal trends in vital signs and complete blood count markers of COVID-19 cases and controls. All mixed-models were adjusted for age. *P < 0.005.

At presentation, COVID-19 cases were more likely to be asymptomatic, compared with the controls (161 of 326 [49.4%] versus 111 of 597 [18.6%]; *P* < 0.0001) (Table 2). COVID-19 cases who were symptomatic, complained mainly of fever (107 of 326 [32.8%]), coryza (57 of 326 [17.5%)], and cough (55 of 326 [16.9%]). Anosmia was only present in 4 of 326 (1.2%) of cases. Compared with controls, COVID-19 cases were more likely to report sore throat (28 of 326 [8.6%] versus 18 of 597 [3.0%]; *P* = 0.0002) and headache (10 of 326 [3.1%] versus 7 of 597 [1.2%]; *P* = 0.0407). Vital signs recorded throughout the hospital stay, demonstrate a lower heart rate, higher blood pressure and higher oxygen saturation in COVID-19 cases compared with controls after adjusting for age (Figure 2). Inpatients with available laboratory investigations are presented in Table 3. Compared with controls, hemoglobin was higher and white cell count was lower in COVID-19 cases, likely due to the lower neutrophil count (3.1 [2.2–4.2] versus 7.8 [4.0, 11.7] × 10^9^/L; *P* < 0.0001) (Figure 2). Serum albumin was higher and total bilirubin, aspartate aminotransferase, and alanine aminotransferase were lower in the COVID-19 cases compared with controls. Only 2 or 326 (0.6%) COVID-19 cases exhibited hepatic organ dysfunction in the form of raised aspartate aminotransferase (range 63–104 U/L) and alanine aminotransferase (range 104–202 U/L); these were mild cases.

**Table 2:** Summary of clinical symptoms at presentation in COVID-19 cases and controls

Clinical symptoms	COVID-19 (*N* = 326)	Control (*N* = 597)	Total (*N* = 932)	*P* value
Asymptomatic	161 (49.4)	111 (18.6)	272 (29.2)	< 0.0001
Fever	107 (32.8)	263 (44.1)	370 (40.1)	0.0009
Cough	55 (16.9)	395 (66.2)	450 (48.8)	< 0.0001
Coryza	57 (17.5)	274 (45.9)	331 (35.9)	< 0.0001
Sore throat	28 (8.6)	18 (3.0)	46 (5.0)	0.0002
Wheezing	0 (0.0)	108 (18.1)	108 (11.7)	< 0.0001
Crepitations	0 (0.0)	33 (5.5)	33 (3.6)	< 0.0001
Headache	10 (3.1)	7 (1.2)	17 (1.8)	0.0407
Myalgia	3 (0.9)	3 (0.5)	6 (0.7)	0.4504
Irritability	2 (0.6)	2 (0.3)	4 (0.4)	0.5381
Refuse feeding	1 (0.3)	41 (6.9)	42 (4.6)	< 0.0001
Diarrhea	13 (4.0)	38 (6.4)	51 (5.5)	0.1308
Vomiting	6 (1.8)	89 (14.9)	95 (10.3)	< 0.0001

Variables are expressed as counts (percentages). *P* values are based on chi-square test.

**Table 3:** Summary of laboratory data in COVID-19 cases and controls

Laboratory parameters	Data available	COVID-19 (*N* = 326)	Control (*N* = 597)	Total (*N* = 932)	*P* value
Hemoglobin, g/dL	412	13.1 (12.4–13.8)	12.5 (11.7–13.4)	12.7 (11.9–13.7)	0.0001
WBC, ×109/L	412	8 (6.4–9.7)	13.3 (9.9–16.9)	10.9 (7.8–15.1)	< 0.0001
Lymphocyte, ×109/L	397	3.3 (2.5–5.2)	3.3 (2.2–5.8)	3.3 (2.4–5.6)	0.5445
Neutrophil, ×109/L	397	3.1 (2.2–4.2)	7.8 (4.0–11.7)	4.7 (2.6–9.4)	< 0.0001
Platelets, ×109/L	410	325 (276–378)	333 (264–414)	330 (269–401)	0.4786
Total protein, g/dL	150	74 (70–78)	72 (67–78)	74 (70–78)	0.0886
Albumin, g/dL	213	41 (39–43)	38 (34–43)	41 (38–43)	0.0111
Total bilirubin, umol/L	211	6 (5,8)	7.5 (5–13.5)	7 (5,9)	0.0055
AST, U/L	189	27 (21–36)	37 (26–53)	28 (23–30)	< 0.0001
ALT, U/L	213	16 (13–21)	22 (15–37)	17 (13–25)	< 0.0001
Urea, mmol/L	225	4.0 (3.4–5.3)	3.7 (2.9–4.5)	3.7 (2.9–4.6)	0.2744
Sodium mmol/L	231	140 (139–141)	139 (137–140)	139 (137–140)	0.4501
Potassium, mmol/L	230	4.0 (3.9–4.4)	4.0 (3.5–4.4)	4.0 (3.6–4.4)	0.2174
Creatinine, umol/L	230	34.0 (26.0–48.0)	25.0 (20.0–32.0)	25.5 (20.0–33.0)	0.0458
C-reactive protein, mg/L	191	1.8 (0.2–12.3)	9.4 (1.4–21.1)	8.8 (1.0–18.4)	0.0108

ALT = alanine aminotransferase; AST = aspartate aminotransferase; WBC = white blood cells. Variables are expressed as median (interquartile range). *P* values are based on Mann-Whitney *U* test.

The most common respiratory outcome in COVID-19 cases on discharge was upper respiratory tract infection (64 of 326 [19.7%]), whereas only 0.6% were diagnosed with pneumonia (Table 4). There were three critical and one moderate cases (Supplemental Material, Table 2). Although none of them were on baseline respiratory support, the majority of these patients (3 of 4 [75%]) had an underlying comorbid condition (leukemia, neurogenetic disorder, history of congenital diaphragmatic hernia). One patient presented with septic shock and was treated as for multisystem inflammatory syndrome in children (MIS-C). One patient developed a pneumothorax; this case was labeled as moderate severity because his oxygen saturations on room air was > 94%. For controls, the most common respiratory diagnosis on discharge was also upper respiratory tract infection (106/597 [17.9%]), but a significant proportion were diagnosed with pneumonia (63/597 [10.6%]). A higher proportion of controls required respiratory support, but there were no major differences in terms of organ dysfunction. During admission, 26 of 597 (4.4%) control cases required intensive care compared with 4 or 326 (1.2%) COVID-19 cases.

**Table 4:** Summary of clinical outcomes in COVID-19 cases and controls

Outcomes	COVID-19 (*N* = 326)	Control (*N* = 597)	Total (*N* = 932)	*P* value
Severity				
Mild	321 (98.5)	498 (84.0)	819 (87.8)	< 0.0001
Moderate	1 (0.3)	70 (11.8)	71 (7.6)	
Severe	0 (0.0)	22 (3.7)	22 (2.4)	
Critical	3 (0.9)	3 (0.5)	6 (0.6)	
Respiratory diagnosis on discharge				
URTI	64 (19.7)	106 (17.9)	170 (18.5)	0.4979
Bronchitis/bronchiolitis	0 (0.0)	1 (0.3)	130 (14.2)	< 0.0001
Pneumothorax	1 (0.3)	1 (0.2)	2 (0.2)	0.6656
Pneumonia	2 (0.6)	63 (10.6)	65 (7.1)	< 0.0001
Respiratory support				
Oxygen therapy	2 (0.6)	51 (8.6)	53 (5.8)	< 0.0001
HFNC	0 (0.0)	6 (1.0)	6 (0.7)	0.0689
CPAP	0 (0.0)	8 (1.3)	8 (0.9)	0.0355
BiPAP	1 (0.3)	10 (1.7)	11 (1.2)	0.0664
Mechanical ventilation	1 (0.3)	10 (1.7)	11 (1.2)	0.0664
Organ dysfunction				
Cardiovascular	1 (0.3)	4 (0.7)	5 (0.5)	0.4702
Respiratory	2 (0.6)	4 (0.7)	6 (0.6)	0.932
Neurological	0 (0.0)	3 (0.5)	3 (0.3)	0.1990
Hepatic	2 (0.6)	1 (0.2)	3 (0.3)	0.2567
Renal	0 (0.0)	3 (0.5)	3 (0.3)	0.1990
Hematological	1 (0.3)	2 (0.3)	3 (0.3)	0.9401
Highest inpatient status				
General ward	322 (98.8)	563 (94.3)	885 (95.9)	0.0038
Intermediate care	0 (0.0)	8 (1.3)	8 (0.9)	
Intensive care	4 (1.2)	26 (4.4)	30 (3.3)	
Hospital duration, days	8 (3,12)	2 (1,2)	2 (1,7)	< 0.0001
Mortality	0 (0.0)	4 (0.7)	4 (0.4)	0.2592

BiPAP = bilevel positive airway pressure; CPAP = continuous positive airway pressure; HFNC = high-flow nasal cannula; URTI = Upper respiratory tract infection. Variables are expressed as counts (percentages). *P* values are based on chi-square test.

Eight COVID-19 cases (2.5%) had coinfections (Table 5). The most common viral coinfection was rhinovirus (*n* = 3). In comparison, control cases consisted of viral (157 of 597 [26.3%]) and bacterial infections (32 of 597 [5.4%]) from various sources. In the multivariable analysis, older age (aOR 1.2 [95% CI: 1.1–1.2]; *P* < 0.001), travel history (aOR 1.7 [95% CI: 1.1–2.6); *P* = 0.0267) and close contact with an infected household member (aOR 12.9 [95% CI: 8.7–19.0]; *P* < 0.001) were associated with COVID-19 infection (Table 6).

**Table 5:** Viruses and bacteria identified in COVID-19 and control cases

Coinfections		COVID-19 cases	Controls
Viruses	Rhinovirus	3	123
	Parainfluenza (3 + 4)		5
	Adenovirus		5
	Respiratory syncytial virus	1	7
	Influenza A		2
	Metapneumovirus		1
	> 1 respiratory virus	2	9
	Dengue virus		3
	Rotavirus		1
	Varicella zoster virus		1
Bacteria	*Staphylococcus aureus*	1	5
	*Streptococcus pneumoniae*		2
	Moraxella *catarrhalis*		2
	*Escherichia coli*		6
	*Pseudomonas aeruginosa*	1	6
	*Klebsiella pneumoniae*		2
	*Enterobacter cloacae*		1
	*Acinetobacter ursingii*		1
	*Salmonella*		1
	Mycoplasma		1
	*Chlamydia*		1
	> 1 bacteria		4

**Table 6:** Multivariable regression model for COVID-19 infection

Covariates	Univariate		Multivariate	
	OR (95% CI)	*P* value	aOR (95% CI)	*P* value
Age	1.1 (1.1–1.2)	< 0.0001	1.2 (1.1–1.2)	< 0.0001
Travel (ref: none)	3.9 (2.8–5.5)	< 0.0001	1.7 (1.1–2.6)	0.0267
Household contact (ref: none)	14.1 (10.1–19.7)	< 0.0001	12.9 (8.7–19.0)	< 0.0001
Sore throat (ref: none)	3.0 (1.6–5.6)	0.0004	1.9 (0.8–4.5)	0.1194
Comorbidity (ref: none)	0.3 (0.2–0.4)	< 0.0001	0.5 (0.3–0.8)	0.0020

aOR = adjusted odds ratio; CI = confidence interval; OR = odds ratio; ref = reference. Results did not change when year of infection (2020 vs. 2021) was added to the regression model.

There were 216 of 326 (66.3%) cases identified in 2020 and 110 of 326 (33.7%) in 2021 (Supplemental Material, Tables 3–6). The 2021 COVID-19 cases were associated less with exposure to infected household contacts (78/110 [70.9%] versus 182 of 216 [83.8%]); *P* = 0.0065) and had shorter hospital stays (3.0 [2.0–4.0] versus 9.0 [7.0–15.5] days; *P* ≤ 0.0001). However, a 2021 year of admission seemed to be associated with greater severity of COVID-19 infection (moderate–critical severity) and ICU admission. Clinical presentation and laboratory markers were similar between the two cohorts. With year of infection included in the multivariable model, there was no significant differences in overall results and the risk of getting infected with SARS-CoV2 in children did not change between 2020 and 2021.

## DISCUSSION

This test-negative case–control study of COVID-19 in children from two neighboring Southeast Asian countries, Singapore and Malaysia, found that pediatric COVID-19 was mostly mild compared with control cases presenting with suspected SARS-CoV-2 infection. They were more likely to be asymptomatic and less likely to develop lower respiratory/pulmonary disease or require respiratory support, to develop organ dysfunction, or require higher level of inpatient care. Besides the association of a lower neutrophil count, COVID-19 cases were less likely to have derangements in laboratory markers. In the multivariable analysis, older age, travel history, and close contact with an infected household member were associated with COVID-19 infection in Singapore and Malaysia.

The proportion of COVID-19 cases in hospitalized children presenting without symptoms (approximately 50%) were consistent with published reports. Some children who met epidemiological criteria were admitted to the hospital for isolation purposes. At the beginning of the pandemic, when the clinical course in children was unknown, patients may have been admitted for monitoring, even though they were asymptomatic. As the pandemic evolved, asymptomatic children were only admitted if they did not have suitable conditions for home isolation. Similar to studies from other regions, the common presenting symptoms of COVID-19 in our cohort were fever (32.8%), coryza (17.5%), and cough (16.9%). In terms of laboratory parameters, when comparing children with COVID-19 with the control group, COVID-19 cases tended to have higher hemoglobin and albumin but lower white cell count—specifically, neutrophil count, total bilirubin, aspartate aminotransferase, and alanine aminotransferase. We found that COVID-19 cases in children tended to have lower heart rate, higher blood pressure, and higher oxygen saturation compared with controls even after adjusting for age (Figure 2). This finding may suggest that the physiological impact of COVID-19 in children could be less than other common etiologies of respiratory infections in children. Additional, larger studies in other settings are needed to confirm this.

The source of viral exposure in this study was consistent with that reported in the literature, with most cases reported to have had contact with an infected household member (259 or 326 [79.4%]). ^29^^,^ ^30^ However, it was observed that the proportion exposed to an identified infected case was lower in 2021 compared with 2020 (85 of 110 [77.2%] versus 186 of 216 [87.3%]; *P* = 0.0198), possibly signifying a trend toward greater spread of unlinked cases in the two countries. Another observed trend was the shortened duration of hospitalization in COVID-19 cases in 2021 (3 [2–4] versus 9 [7–15.5] days; *P* < 0.0001), reflecting transfer of care to other isolation facilities or isolation at home. ^31^ However, there were patients with greater severity of illness presenting in 2021. Of note, two of four (50%) of these had coinfections (Supplemental Table 2). We were also not able to ascertain whether these were due to COVID-19 variants of concern because of the absence of strain sequencing data, although surveillance data suggest increased introduction and transmission of SARS-CoV-2 delta variant in 2021. ^32^^,^ ^33^ Studies in adults have reported the SARS-CoV-2 delta variant to be associated with increased transmission and disease severity. ^34^ In children, the delta variant was associated with increased COVID-19–associated hospitalization, although proportion of severe disease was not different. ^35^

Four COVID-19 cases had coinfection with rhinovirus (three) and respiratory syncytial virus (RSV; one). In controls, rhinovirus (123) was identified as the main causative viral pathogen. This was followed by RSV (seven), Adenovirus (five), parainfluenza (five), and influenza (two). The decrease in typical viral circulation was likely due to pandemic control measures, including lockdowns and school closures as reported in other countries; however, rhinovirus seems to have persisted. There were three dengue cases detected in the control arm, demonstrating ongoing transmission of vectorborne diseases among children during the pandemic. There have been reports of COVID-19 cases wrongly diagnosed as dengue in adults and clinicians in dengue endemic countries need to remain vigilant of this risk in children. A significant number of bacterial pathogens from various sources were identified in the control arm highlighting the difficulty in distinguishing COVID-19 infections from other infections in children based on clinical presentation alone. Despite the ongoing pandemic, prescription of appropriate antibiotics should not be delayed for suspected unwell pediatric cases while awaiting laboratory confirmation of COVID-19.

This study incorporated clinical registry data from the two largest pediatric centers in Singapore and Malaysia. Patient identification was complete and datasets had few missing data; hence, the capture population were representative of these cities’ pediatric population. However, it may not be representative of more rural towns in Malaysia or other countries in Southeast Asia. School closures were not uniform between the two countries and the proportion of children attending school in the weeks preceding admission was undetermined; therefore, this study is unable to provide data on school transmission. We were not able to identify patients according to their admission criteria (clinical or epidemiological) or whether COVID-19 diagnosis was incidental. This would have provided clearer information on prognosis/outcomes. Although detailed clinical and laboratory data were available on patients who tested positive for COVID-19, we were not able to determine whether the severe/critical cases were solely due to SARS-CoV2. Moreover, there were rapidly evolving viral factors (e.g., emerging variants of concern), host factors (e.g., transmission patterns, vaccination status), and community-based mitigation measures over the period of study, which we were unable to account for. Another limitation of this study is that the data gathered were limited to the duration of hospital stay without any longer term follow-up. Hence, intermediate to long-term complications were not captured. Lastly, our analysis of differences by year of infections was a proxy measure because we did not have data on individual COVID-19 viral strains.

## CONCLUSION

Although some children with COVID-19 could have severe clinical manifestations, the vast majority of pediatric COVID-19 cases in the Singapore and Malaysia within the first 15 months of the pandemic were mild and of lesser severity compared with other common community-acquired infections in children. During the course of infection, pediatric COVID-19 cases consistently trended high hemoglobin and platelets but low neutrophil count compared with controls. Older age, travel history, and household exposure to an infected COVID-19 case were associated with COVID-19 infection.

## Supplemental Material


Supplemental materials

## References

[1] WHO , 2021. Coronavirus Disease (COVID-19), 2021. Technical Guidance. Geneva, Switzerland: World Health Organization. Available at: https://www.who.int/emergencies/diseases/novel-coronavirus-2019/technical-guidance/naming-the-coronavirus-disease-(covid-2019)-and-the-virus-that-causes-it. Accessed July 5, 2021.

[2] Goh T Toh TW , 2020. Singapore confirms first case of Wuhan virus; second case likely. The Straits Times. 2020: 23.

[3] 2020. First coronavirus cases in Malaysia: 3 Chinese nationals confirmed infected, quarantined in Sungai Buloh Hospital. Benama. 2020: 25.

[4] United Nations , 2020. *Policy Brief: The Impact of COVID-19 on South-East Asia.* Available at: https://unsdg.un.org/resources/policy-brief-impact-covid-19-south-east-asia.

[5] Lee WC Ong CY , 2020. Overview of rapid mitigating strategies in Singapore during the COVID-19 pandemic. Public Health 185: 15–17. 32516621 10.1016/j.puhe.2020.05.015 PMC7242914

[b6] Danial M 2020. Mitigation of COVID-19 clusters in Malaysia. J Glob Health 10: 0203105. 33403108 10.7189/jogh.10.0203105 PMC7750020

[b7] Muhamad Khair NK Lee KE Mokhtar M , 2021. Community-based monitoring in the new normal: a strategy for tackling the COVID-19 pandemic in Malaysia. Int J Environ Res Public Health 18: 6712. 34206384 10.3390/ijerph18136712 PMC8297202

[8] Teoh S , 2021. Malaysia’s King declares state of emergency till Aug 1 to curb spread of COVID-19. The Straits Times 2021: 12.

[9] Cruz AT Zeichner SL , 2020. COVID-19 in children: initial characterization of the pediatric disease. Pediatrics 145: e20200834. 32179659 10.1542/peds.2020-0834

[10] Dong Y 2020. Epidemiology of COVID-19 among children in China. Pediatrics 145: e20200702. 32179660 10.1542/peds.2020-0702

[11] Tezer H Bedir Demirdağ T , 2020. Novel coronavirus disease (COVID-19) in children. Turk J Med Sci 50: 592–603. 32304191 10.3906/sag-2004-174 PMC7195991

[12] Yonker LM 2020. Pediatric severe acute respiratory syndrome coronavirus 2 (SARS-CoV-2): clinical presentation, infectivity, and immune responses. J Pediatr 227: 45–52.e5. 32827525 10.1016/j.jpeds.2020.08.037 PMC7438214

[b13] Bunyavanich S Do A Vicencio A , 2020. Nasal gene expression of angiotensin-converting enzyme 2 in children and adults. JAMA 323: 2427–2429. 32432657 10.1001/jama.2020.8707 PMC7240631

[14] Muus C 2020. Integrated analyses of single-cell atlases reveal age, gender, and smoking status associations with cell type-specific expression of mediators of SARS-CoV-2 viral entry and highlights inflammatory programs in putative target cells. bioRxiv Available at: 10.1101/2020.04.19.049254 .

[15] Zhou F 2020. Clinical course and risk factors for mortality of adult inpatients with COVID-19 in Wuhan, China: a retrospective cohort study. Lancet 395: 1054–1062. 32171076 10.1016/S0140-6736(20)30566-3 PMC7270627

[16] Viner RM 2021. Susceptibility to SARS-CoV-2 infection among children and adolescents compared with adults: a systematic review and meta-analysis. JAMA Pediatr 175: 143–156. 32975552 10.1001/jamapediatrics.2020.4573 PMC7519436

[17] Kam KQ 2021. SARS-CoV-2 viral RNA load dynamics in the nasopharynx of infected children. Epidemiol Infect 149: e18. 33427152 10.1017/S095026882100008X PMC7847743

[18] Macartney K 2020. Transmission of SARS-CoV-2 in Australian educational settings: a prospective cohort study. Lancet Child Adolesc Health 4: 807–816. 32758454 10.1016/S2352-4642(20)30251-0 PMC7398658

[b19] Heavey L 2020. No evidence of secondary transmission of COVID-19 from children attending school in Ireland, 2020. Euro Surveill 25: 2000903. 10.2807/1560-7917.ES.2020.25.21.2000903 PMC7268273 32489179

[b20] Yung CF 2020. Novel coronavirus 2019 transmission risk in educational settings. Clin Infect Dis 72: 1055–1058. 10.1093/cid/ciaa794 PMC7337629 32584975

[21] Budzyn SE 2021. Pediatric COVID-19 cases in counties with and without school mask requirements—United States, July 1–September 4, 2021. MMWR Morb Mortal Wkly Rep 70: 1377–1378. 34591829 10.15585/mmwr.mm7039e3 PMC8486393

[22] Wong JJM 2021. Comparative analysis of pediatric COVID-19 infection in Southeast Asia, South Asia, Japan, and China. Am J Trop Med Hyg. 105: 413–420. 34129517 10.4269/ajtmh.21-0299 PMC8437183

[b23] George S 2021. COVID-19 in children in Brunei Darussalam: higher incidence but mild manifestations. J Med Virol 93: 199–201. 32687229 10.1002/jmv.26310 PMC7405023

[24] Ng DC 2021. Clinical and epidemiological characteristics of children with COVID-19 in Negeri Sembilan, Malaysia. Int J Infect Dis 108: 347–352. 34087485 10.1016/j.ijid.2021.05.073 PMC8168297

[25] Deeb A 2021. Impact of ethnicity and underlying comorbidity on COVID-19 inhospital mortality: an observational study in Abu Dhabi, UAE. BioMed Res Int 2021: 6695707. 33708993 10.1155/2021/6695707 PMC7930915

[26] Harris PA 2009. Research electronic data capture (REDCap)—a metadata-driven methodology and workflow process for providing translational research informatics support. J Biomed Inform 42: 377–381. 18929686 10.1016/j.jbi.2008.08.010 PMC2700030

[27] von Elm E 2007. The Strengthening the Reporting of Observational Studies in Epidemiology (STROBE) statement: guidelines for reporting observational studies. Prev Med 45: 247–251. 17950122 10.1016/j.ypmed.2007.08.012

[28] World Health Organization , 2020. Clinical Management of COVID-19—Interim Guidance. Geneva, Switzerland: WHO. Available at: https://apps.who.int/iris/handle/10665/332196.

[29] Du W 2020. Clinical characteristics of COVID-19 in children compared with adults in Shandong Province, China. Infection 48: 445–452. 32301099 10.1007/s15010-020-01427-2 PMC7161094

[30] Bi Q 2020. Epidemiology and transmission of COVID-19 in 391 cases and 1286 of their close contacts in Shenzhen, China: a retrospective cohort study. Lancet Infect Dis 20: 911–919. 32353347 10.1016/S1473-3099(20)30287-5 PMC7185944

[31] Lim MZ Yuen S, 2021. 2,400 bed spaces for COVID-19 patients at community care facilities, 1,700 more as buffer: MOH. *The Straits Times*, May 25.

[32] Tan A , 2021. 550 COVID-19 cases infected with delta variant detected in Singapore so far. *The Straits Times*, June 10, 2021. Available at: https://www.straitstimes.com/singapore/health/550-out-of-about-62000-covid-19-cases-in-singapore-infected-with-delta-variant.

[33] Kenyataan Akhbar KPK , 2021. *Situasi Semasa Jangkitan Penyakit Coronavirus 2019 (COVID-19) di Malaysia: Director General of Health.* Available at: https://kpkesihatan.com/2021/06/20/kenyataan-akhbar-kpk-20-jun-2021-situasi-semasa-jangkitan-penyakit-coronavirus-2019-covid-19-di-malaysia/. Accessed June 20, 2021.

[34] Ong SWX 2021. Clinical and virological features of SARS-CoV-2 variants of concern: a retrospective cohort study comparing B.1.1.7 (alpha), B.1.315 (beta), and B.1.617.2 (delta). Clin Infect Dis 20: 911–919. 10.1093/cid/ciab721 PMC8522361 34423834

[35] Delahoy M Ujamaa D Whitaker M , 2021. Hospitalizations Associated with COVID-19 Among Children and Adolescents — COVID-NET, 14 States, March 1, 2020–August 14, 2021. MWR Morb Mortal Wkly Rep. Available at: 10.1093/cid/ciab721. PMC8437052 34499627

