# Supplementary material for: Pediatric COVID-19 Risk Factors in Southeast Asia-Singapore and Malaysia: A Test-Negative Case–Control Study
Source: Am J Trop Med Hyg. 2022 Feb 15;106(4):1113–20. doi: 10.4269/ajtmh.21-1000 (PMC8991357; doi:10.4269/ajtmh.21-1000)
Supplement: Supplementary file 1 [file tpmd211000.SD1.pdf]

Supplemental Tables

Supplemental Table 1: National screening criteria in Singapore and Malaysia

Country	Singapore: KK Women and Children's Hospital	Malaysia: University Malaya Medical Center
Clinical criteria	• Community-acquired pneumonia<br>• Acute respiratory infection or fever<br>• Symptomatic patients requiring clinical admission<br>• Clinical features suggestive of Kawasaki Disease/ hemophagocytic lymphohistiocytosis/ macrophage activation syndrome<br>• Septic/Infectious condition needing high dependency/ intensive care	• Respiratory symptoms such as fever, chills, sore throat, nausea, cough, shortness of breath etc<br>• Pneumonia or acute respiratory distress syndrome
Epidemiological criteria	• Close contact with a positive case<br>• Any travel<br>• Stayed in foreign worker dormitory<br>• Work in environments with high exposure to COVID-19 cases	• Attended an event in areas associated with known COVID-19 cluster or red zones<br>• Travelled to or resided in a foreign country within 14 days before the onset of illness<br>• Close contact to a confirmed case of COVID-19, within 14 days before the onset of illness

NB: Suspect criteria was met when any one of the clinical or epidemiological criteria were present

Supplemental Table 2: Characteristics of severe/critical COVID-19 cases

**Patient ID**	**379-750**	**379-1211**	**379-1212**	**379-1213**
Gender	Male	Male	Male	Female
Age, years	16	1.5	3	4.8
Comorbidity	History of left congenital diaphragmatic hernia and chronic lung disease	None	Acute myeloid leukemia post-hematopoietic stem cell transplant	Neurogenetic disorder
Baseline respiratory support	None	None	None	None
Clinical features	Chest pain	Fever, coryza, severe stridor, cyanosis, hypoxic seizure (GTC), diarrhea, erythematous macular popular rash (upper limbs), pustular rash (neck)	Fever, vomiting, septic shock	Respiratory distress
CXR	Left pneumothorax	Right perihilar opacification	Clear	Bilateral consolidation
COVID-19 test	RT-PCR positive (CT 35.53)	RT-PCR positive (CT 34.42)<br>IgM and IgG weakly positive	RT-PCR positive (CT 33.77)	RT-PCR positive (CT 19.50)
Co-infection	None	Respiratory syncytial virus (respiratory PCR)<br>Methicillin sensitive *Staphylococcus aureus* (tracheal aspirate culture)	*Pseudomonas aeruginosa* (blood culture)	None
Treatment	Face mask oxygen 10L/min	Nebulized budesonide<br>Nebulized adrenaline<br>Systemic corticosteroids<br>Mechanical ventilation<br>IV ceftriaxone and cloxacillin	Fluid resuscitation<br>IV noradrenaline infusion<br>IV meropenem and piperacillin-tazobactam	BiPAP<br>IV co-amoxiclav
Outcome	Recovered	Recovered	Recovered	Recovered

BiPAP – bilevel positive airway pressure, CT – cycle threshold, CXR – chest X-ray, IV-intravenous, RT-PCR – reverse transcriptase-polymerase chain reaction,

Supplemental Table 3: Summary of demographic data comparing COVID-19 cases in 2020 and 2021

Demographics	COVID-19 2020 (n=216)	COVID-19 2021 (n=110)	Total (n=326)	P value
Age, years	6.0 (2.5, 12.0)	8.0 (4.0, 11.0)	7.0 (3.0, 12.0)	0.1376
Weight, kg	20.3 (11.5, 43.6)	21.1 (12.0, 35.6)	20.6 (11.8, 37.8)	0.6108
Male gender	115 (53.2)	53 (38.2)	168 (51.5)	0.3875
Infants	14 (6.5)	7 (6.4)	21 (6.4)	0.9673
Travel history	107 (49.5)	54 (49.5)	161 (49.5)	0.9994
Exposure to confirmed case	186 (87.3)	85 (77.2)	271 (83.9)	0.0198
Healthcare	4 (1.9)	1 (0.9)	5 (1.5)	0.5125
School	2 (0.9)	0 (0.0)	2 (0.6)	0.3114
Household	182 (83.8)	78 (70.9)	259 (79.4)	0.0065
Others	4 (1.9)	8 (7.3)	12 (3.7)	0.0140
Comorbidity	26 (18.0)	6 (5.5)	32 (9.8)	0.0655
Cardiovascular	2 (0.9)	0 (0.0)	2 (0.6)	
Respiratory	4 (1.9)	2 (1.8)	6 (1.8)	
Neurology	2 (0.9)	0 (0.0)	2 (0.6)	
Hematology/ Oncology	1 (0.5)	3 (2.7)	4 (1.2)	
Renal	0 (0.0)	0 (0.0)	0 (0.0)	
Gastrointestinal	1 (0.5)	0 (0.0)	1 (0.3)	
Others	16 (7.4)	1 (0.9)	17 (5.2)	

Note: categorical and continuous variables are expressed as counts (percentages) and median (interquartile range) respectively. P –values are based on Chi-Square test and Mann-Whitney U – test for categorical and continuous variables respectively.

Supplemental Table 4: Summary of clinical symptoms at presentation comparing COVID-19 cases in 2020 and 2021

Clinical symptoms	COVID-19 2020 (n=216)	COVID-19 2021 (n=110)	Total (n=326)	P value
Asymptomatic	104 (48.2)	57 (51.8)	161 (49.4)	0.5309
Fever	71 (32.9)	36 (32.7)	107 (32.8)	0.9792
Cough	37 (17.1)	18 (16.4)	55 (16.9)	0.8614
Coryza	41 (19.0)	16 (14.5)	57 (17.5)	0.3187
Sore throat	19 (8.8)	9 (8.2)	28 (8.8)	0.8515
Wheezing	0 (0.0)	0 (0.0)	0 (0.0)	-
Crepitations	0 (0.0)	0 (0.0)	0 (0.0)	-
Headache	2 (0.9)	0 (0.0)	2 (0.6)	0.3114
Myalgia	1 (0.5)	2 (1.8)	3 (0.9)	0.2256
Irritability	2 (0.9)	0 (0.0)	2 (0.6)	0.3114
Refuse feeding	1 (0.5)	0 (0.0)	1 (0.5)	0.4748
Diarrhea	9 (4.2)	4 (3.6)	13 (4.0)	0.8170
Vomiting	3 (1.4)	3 (2.7)	6 (1.8)	0.8170

Note: variables are expressed as counts (percentages). P –values are based on Chi-Square test.

Supplemental Table 5: Summary of clinical outcomes comparing COVID-19 cases in 2020 and 2021

Outcomes	COVID-19 2020 (n=216)	COVID-19 2021 (n=110)	Total (n=326)	P value
Severity				
Mild	215 (99.5)	106 (96.4)	321 (98.5)	0.0127
Moderate	0 (0.0)	1 (0.9)	1 (0.3)	
Severe	0 (0.0)	0 (0.0)	0 (0.0)	
Critical	0 (0.0)	3 (2.7)	3 (0.9)	
Respiratory diagnosis				
Upper respiratory tract infection	61 (28.4)	3 (2.7)	64 (19.7)	<0.0001
Bronchitis/bronchiolitis	0 (0.0)	0 (0.0)	0 (0.0)	-
Pneumothorax	0 (0.0)	1 (0.9)	1 (0.3)	0.1615
Pneumonia	0 (0.0)	2 (1.8)	2 (0.6)	0.0473
Respiratory support				
Oxygen therapy	1 (0.5)	1 (0.9)	2 (0.8)	0.6282
HFNC	0 (0.0)	0 (0.0)	0 (0.0)	-
CPAP	0 (0.0)	0 (0.0)	0 (0.0)	-
BiPAP	0 (0.0)	1 (0.9)	1 (0.3)	0.1615
Mechanical ventilation	0 (0.0)	1 (0.9)	1 (0.3)	0.1615
Organ dysfunction				
Cardiovascular	0 (0.0)	1 (0.9)	1 (0.3)	0.1615
Respiratory	0 (0.0)	2 (1.8)	2 (0.6)	0.0470
Neurological	0 (0.0)	0 (0.0)	0 (0.0)	-
Hepatic	2 (0.9)	0 (0.0)	2 (0.6)	0.3103
Renal	0 (0.0)	0 (0.0)	0 (0.0)	-
Hematological	0 (0.0)	1 (0.9)	1 (0.3)	0.1615
Highest inpatient status				
General ward	216 (100.0)	106 (96.4)	322 (98.8)	0.0048
Intermediate care	0 (0.0)	0 (0.0)	0 (0.0)	
Intensive care	0 (0.0)	4 (3.6)	4 (1.2)	
Hospital duration, days	9.0 (7.0, 15.5)	3.0 (2.0, 4.0)	8.0 (3.0, 11.0)	<0.0001
Mortality	0 (0.0)	0 (0.0)	0 (0.0)	-

Note: variables are expressed as counts (percentages). P –values are based on Chi-Square test. HFNC – high flow nasal cannula, CPAP – continuous positive airway pressure, BiPAP – bilevel positive airway pressure

Supplemental Table 6: Summary of laboratory data comparing COVID-19 cases in 2020 and 2021

Laboratory parameters	Data available	COVID-19 2020 (n=216)	COVID-19 2021(n=110)	Total (n=326)	P value
Hemoglobin, g/dL	148	13.1 (12.4, 13.8)	12.6 (11.7, 13.3)	13.1 (12.4, 13.8)	0.1022
WBC, x109/L	148	8.0 (6.4, 10.1)	8.4 (6.5, 8.8)	8.0 (6.4, 9.7)	0.5554
Lymphocyte, x109/L	148	3.3 (2.5, 5.5)	3.0 (2.1, 3.3)	3.3 (2.5, 5.2)	0.1615
Neutrophil, x109/L	148	3.1 (2.2, 4.2)	3.8 (2.1, 4.4)	3.1 (2.2, 4.2)	0.6274
Platelets, x109/L	148	331 (278, 381)	278 (275, 325)	325 (276, 378)	0.2136
Total protein, g/dL	125	74 (70, 78)	74 (74, 76)	74 (70, 78)	0.8746
Albumin, g/dL	135	41 (39, 43)	42 (41, 43)	41 (39, 43)	0.7029
Total bilirubin, umol/L	135	6 (5, 8)	12 (7, 16)	6 (5, 8)	0.0673
AST, U/L	134	27 (21, 36)	26.5 (25, 27)	27 (21, 36)	0.7183
ALT, U/L	136	16 (13 to 22)	12.5 (9 to 18)	16 (13 to 21)	0.1112
Urea, mmol/L	20	4 (3.2, 5.3)	4 (4, 4)	4 (3.4, 5.3)	1.000
Sodium mmol/L	20	140 (138, 141)	140 (140, 140)	140 (139, 141)	0.8575
Potassium, mmol/L	19	4 (3.9, 4.4)	3 (3, 3)	4 (3.9, 4.4)	0.0966
Creatinine, umol/L	19	33.5 (26, 48)	34 (34, 34)	34 (26, 48)	1.000
C-reactive protein, mg/L	20	1.2 (0.2, 7.8)	60.9 (13.9, 108.0)	1.8 (0.22, 12.3)	0.0583

Note: variables are expressed as median (interquartile range). P –values are based on Mann-Whitney U test.
WBC – white blood cells, AST - aspartate aminotransferase, ALT – alanine aminotransferase
